# RT-QuIC detection of pathological prion protein in subclinical goats following experimental oral transmission of L-type BSE

**DOI:** 10.1186/s13104-021-05859-3

**Published:** 2021-12-07

**Authors:** Alessandra Favole, Maria Mazza, Antonio D’Angelo, Guerino Lombardi, Claudia Palmitessa, Luana Dell’Atti, Giulia Cagnotti, Elena Berrone, Marina Gallo, Tiziana Avanzato, Erika Messana, Loretta Masoero, Pier Luigi Acutis, Daniela Meloni, Franco Cardone, Maria Caramelli, Cristina Casalone, Cristiano Corona

**Affiliations:** 1 S.C. Neuroscienze, Lab. di Neurobiologia Sperimentale, Istituto Zooprofilattico Sperimentale del Piemonte, Liguria e Valle d’Aosta, Via Bologna 148, 10154 Turin, Italy; 2 University of Turin, Grugliasco (Turin), Italy; 3 Istituto Zooprofilattico Sperimentale della Lombardia e dell’Emilia-Romagna, Brescia, Italy; 4 Istituto Superiore di Sanità, Rome, Italy

**Keywords:** Prion, L-BSE, RT-QuIC, Goat, Oral transmission, PrP^sc^, Ultrasensitive detection

## Abstract

**Objective:**

The spread of bovine spongiform encephalopathy (BSE) agent to small ruminants is still a major issue in the surveillance of transmissible spongiform encephalopathies (TSEs). L-type bovine spongiform encephalopathy (L-BSE) is an atypical form of BSE with an unknown zoonotic potential that is transmissible to cattle and small ruminants. Our current knowledge of bovine atypical prion strains in sheep and goat relies only on experimental transmission studies by intracranial inoculation. To assess oral susceptibility of goats to L-BSE, we orally inoculated five goats with cattle L-BSE brain homogenates and investigated pathogenic prion protein (PrP^sc^) distribution by an ultrasensitive in vitro conversion assay known as Real-Time Quaking Induced Conversion (RT-QuIC).

**Results:**

Despite a prolonged observation period of 80 months, all these animals and the uninfected controls did not develop clinical signs referable to TSEs and tested negative by standard diagnostics. Otherwise, RT-QuIC analysis showed seeding activity in five out of five examined brain samples. PrP^sc^ accumulation was also detected in spinal cord and lymphoreticular system. These results indicate that caprine species are susceptible to L-BSE by oral transmission and that ultrasensitive prion tests deserve consideration to improve the potential of current surveillance systems against otherwise undetectable forms of animal prion infections.

## Introduction

L-type bovine spongiform encephalopathy (L-BSE) is an atypical form of BSE that is naturally occurring in cattle and experimentally transmissible to small ruminants. Early identification and characterization of TSEs prion strains different from scrapie in sheep and goat is a primary goal of active surveillance as species barrier breakthrough may boost the zoonotic potential of the relevant prion strain.

Experimental transmission by the intracerebral route to sheep and goats showed that both species are susceptible to L-BSE with an efficiency of infection as high as 100% and preferential accumulation of PrP^sc^ in the central nervous system (CNS) and to a lesser extent in the peripheral nervous system, in the cerebrospinal fluid and in the lymphoreticular system (LRS) [1–5].

The intracerebral route offers the possibility to evaluate the species barrier between old and new host, the neuropathogenesis of the disease and the biochemical signature of the prion strain, yet it may not recapitulate the natural history of the disease after oral exposure, by far the main route of possible infection in field conditions.

We here investigate L-BSE transmissibility and PrP^sc^ distribution in orally infected goats.

## Main text

### Methods

#### Animals

Seven, six-month-old goats were purchased from herds with no record of scrapie cases. Five animals were Saanen breed and two were crossbred. Genetic analysis to determine PRNP gene polymorphisms and to exclude subjects carrying mutations that could confer resistance against BSE: L168 and M142 [6, 7] or Scrapie (I142M, N146S/D, R154H, R211Q, Q222K) was performed as previously described [4, 8].

#### Inoculation of goats

PrP^sc^-positive frontal cortex (5 g tissue) from a L-BSE naturally affected cow (IT-141387/02) was ground in a mechanical grinder and homogenized at 30% weight/volume (wt/vol) in sterile buffered saline (PBS). Prior to inoculation, the animals were kept in the new environment for one month for adaptation and were clinically examined to rule out clinical abnormalities. L-BSE inoculum was checked for microbiological sterility and administered to four Saanen and one mixed breed goats (Table 1) by a tube passed through the esophagus down to rumen or abomasum. Two goats were challenged with 15 ml each of PBS and served as controls. The inoculated animals were housed in a bio-safety level 3 containment facility.

**Table 1:** General data of goats experimentally infected with bovine isolates of L-BSE and PrP^sc^-associate seeding activity distribution revealed by RT-QuIC analyses in nervous and lymphoreticular systems of subclinical animals

ID	Inoculum	Route of infection	Breed	Sex	Genotype	Age* (mo)	SV (mo)	BH PrP^sc^	PrP^sc^ peripheral distribution
G1	L-BSE	OS	Mixed Breed	F	240 P/P	6	74	+	LNs: retropharyngeal, submandibular, mesenteric; tonsil; thoracic medulla
G2	L-BSE	OS	Saanen	F	240 P/P	6	45	+	LNs: mesenteric, mediastinal, retropharyngeal, submandibular; tonsil; thoracic medulla
G3	L-BSE	OS	Saanen	F	240 P/P	6	65	+	LNs: submandibular, mesenteric; tonsil; lombar and sacral medulla
G4	L-BSE	OS	Saanen	F	240 P/P	6	60	+	LNs: submandibular, mesenteric, mediastinal, retropharyngeal; tonsil; thoracic medulla
G5	L-BSE	OS	Saanen	F	240 P/P	6	78	+	LNs: mesenteric, mediastinal; tonsil;
CTR +	L-BSE	IC	Saanen	F	240 P/P	6	41	+	CSF
CTR 1	None	-	Mixed Breed	F	240 P/P	6	41	−	Negative
CTR 2	None	-	Saanen	F	240 P/P	6	74	−	Negative

*OS* oral administration, *IC* intracranial, *F* Female, *SV* survival time, *BH* brainstem homogenate, *LNs* Lympho nodes; *mo* months, *n. a.* not available. *Age at inoculation time

#### Clinical evaluation

Daily clinical evaluation was carried out by the animal husbandry staff and the veterinarian. Neurologic examination was performed monthly by a board-certified neurologist. For this purpose, a clinical examination protocol previously used to diagnose scrapie in sheep was applied [9].

#### Tissue sample collection

At various times after exposure, namely 45, 60, 65, 74 and 78 months the animals were anaesthetized with propofol (PropoVet®, Abbott Animal Health) administered intravenously (i.v.), were euthanized with i.v. enbutramide/mebezonium iodide/tetracaine hydrochloride (Tanax®, Intervet Inc. Merck) and were subjected to necroscopy. The whole brain, the cerebrospinal fluid (CSF), the entire spinal cord, the LRS (tonsils, submandibular, retropharyngeal, mesenteric and mediastinal lymph nodes), were sampled. Each sample was divided equally: one half was fixed in 10% buffered formalin and the other one was frozen at − 80 °C.

#### Rapid test

Diagnosis of TSE was performed on the medulla oblongata firstly by an enzyme-linked immunosorbent assay (ELISA)-based method (IDEXX HerdChek BSE-scrapie antigen test kit EIA rapid test—IDEXX Laboratories) followed by a confirmatory western blot method. The CEA internal western blot technique has been described elsewhere [10]. These methods represent the gold standard techniques for making the diagnosis of TSE in CNS samples [11, 12].

#### Pathology and immunohistochemistry

After fixation, the brain of each goat was coronally cut at the level of obex, medulla, pons, cerebellum, midbrain, diencephalon, and telencephalon. The sections were processed and embedded in paraffin and stained with haematoxylin and eosin (H&E). Immunohistochemical (IHC) analysis and evaluation of PrP^sc^ deposition and PrP^sc^ distribution patterns were performed as previously described [4] at the level of obex, rostral medulla, cerebellar vermis, midbrain, thalamus and frontal area.

#### Tissues samples preparation for RT-QuIC analysis

Pools of brain areas was obtained mixing same amount (10 mg) of each area separately sampled (brainstem, hypothalamus, cerebellum, basal nuclei, frontal, temporal and occipital cortex). Goat brain homogenates (BHs) (10% wt/vol) were prepared as previously described [5]. Peripheral tissues samples were digested with 0.25% (10% wt/vol) collagenase A (Roche) in PBS/2 mM CaCl2 at 37 °C with 800 rpm overnight continuous shaking, homogenized in Beads Beater and finally clarified at 2000 × *g* for 5 min. The supernatant was collected and frozen at − 80 °C.

#### RT-QuIC analysis

Goat BHs or peripheral tissues homogenates (THs) were serially diluted in 0.1% sodium dodecyl sulfate (SDS, Sigma)–N2 (Gibco)–PBS and subjected to RT-QuIC by using chimeric hamster-sheep (Ha-S) 23-231 and Hamster 90–231 recombinant PrP^Sen^ (rPrP^Sen^) produced as previously described [13]. The RT-QuIC reaction mix was composed of 10 mM phosphate buffer (pH 7.4), 300 mM NaCl, 10 μM Thioflavin T (ThT), 1 mM Ethylenediaminetetraacetic acid (EDTA), and 0.1 mg/ml of rPrP^Sen^. Aliquots of this mix (98 μl) were loaded into each well of a 96-well plate with a clear bottom (Nunc) and seeded with 2 μl of 10-3 to 10-4 BH or TH dilutions. Normal goat TH dilutions were used as negative controls, and 10 − 4 BH dilutions of goat with clinical L-BSE were included as positive controls. The plate was then sealed with a plate-sealer film (Nunc) and incubated at 42 °C or 55 °C in a BMG FLUOstar plate reader with cycles of 1 min of shaking (700 rpm double orbital) and 1 min of rest throughout the incubation. ThT fluorescence measurements (excitation, 450 ± 10 nm; emission, 480 ± 10 nm; bottom read) were recorded every 45 min. RT-QuIC reactions were deemed acceptable when the negative controls remained negative for at least 120 h. RT-QuIC CSF assay was performed as reported previously [5].

Data sets were normalized to a percentage of the maximal fluorescence response of the instrument, and the obtained values were plotted against the reaction times. Data are displayed as the average of four technical replicates.

### Results

No animal developed clinical signs of prion encephalopathy prior to euthanasia. All inoculated animals (n = 5) and the uninfected controls (n = 2) tested negative by standard diagnostics performed on brainstem. Also, histological and immunohistochemical analyses performed on the selected brain macro-areas and on all the peripheral tissues sampled resulted negative.

**Fig. 1:**
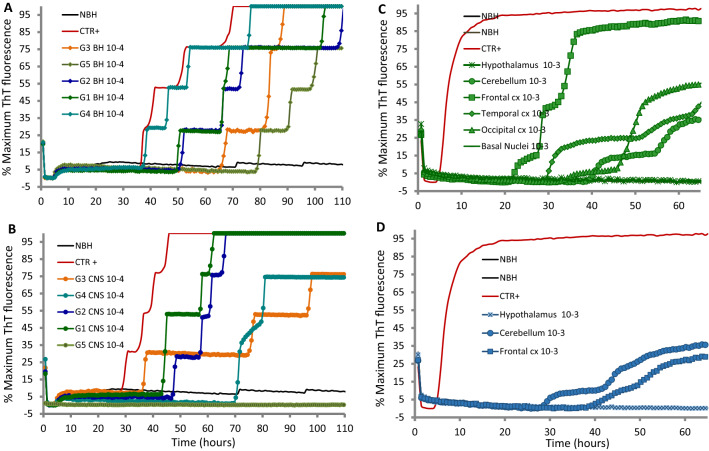
**A**, **B** PrP^sc^ detection from subclinical goats following experimental oral transmission of L-type BSE. rHaPrP^Sen^ 90-231 substrate was used to detect PrP^L-BSE^ from both brainstem **A** and pooled CNS **B** homogenates. Normal control brain homogenates (NBH, black) showed no response. Goat ID and dilutions are indicated next to the curve. C-D) Detection of PrP^sc^ in different CNS areas of L-BSE infected goats at the asymptomatic stage using RT-QuIC assay. rHaSPrP^Sen^ 23-231 substrate was used at 55 °C to detect PrP^L-BSE^ from different CNS areas collected from **C** G1 and **D** G2 goats. Each ThT reading is indicated as the percentage of the maximum value achievable by the plate readers as a function of reaction time

For ultrasensitive PrP^sc^ detection, brainstem and a pool of different brain areas from each animal were homogenized, serially diluted in logarithmic steps in 0.1% SDS–N2–PBS and subjected to RT-QuIC by using chimeric Ha-S 23-231 and Ha 90-231 rPrP^Sen^. Five out of five examined brainstem samples showed positive RT-QuIC reactions (Fig. 1A) with stronger fluorescence increase for G4 goat and an average lag phase of 56 h. Furthermore, PrP^sc^ accumulation was also detected in pooled brain samples of four (G1, G2, G3, G4) out of five examined goats. Normal control brain homogenates (NBH) showed no response (Fig. 1B).

To identify brain areas associated to PrP^sc^ deposition, dilutions of multiple brain regions from G1 and G2 goats were tested: we detected a significant increase in fluorescence and shorter lag phase in cerebral cortex and cerebellum samples but not in basal nuclei and hypothalamus (Fig. 1C, D).

**Fig. 2:**
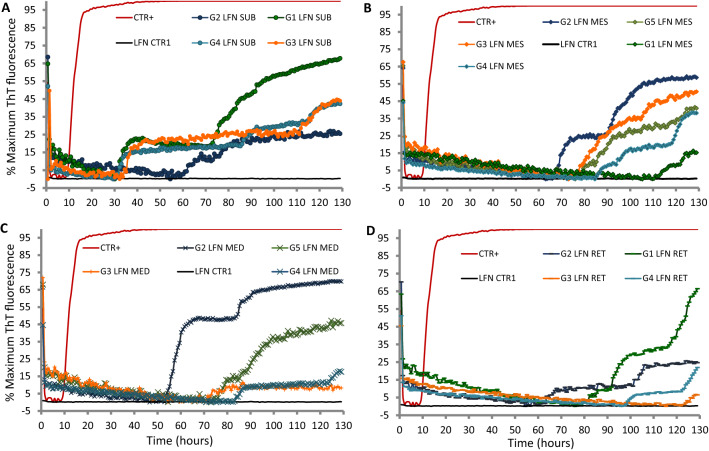
RT-QuIC detection of PrP^sc^ in different lympho nodes (**A** submandibular, **B** mesenteric, **C** mediastinal and **D** retropharyngeal) of L-BSE infected goats. Normal control tissue homogenates (black) showed no response. Goat ID are indicated next to the curve. RT-QuIC reactions were seeded with 2 μl of 10^–4^ LNs homogenate dilution and rHaPrP^Sen^ 29-231 substrate was used at 42 °C to detect PrP^L-BSE^. Each ThT reading is indicated as the percentage of the maximum value achievable by the plate readers as a function of reaction time. The positive threshold was calculated as 10% fluorescence increases of tissue homogenate from normal controls

By using a modified version of RT-QuIC sample homogenization procedure, we also tested PrP^sc^ distribution in the LRS (tonsils, lymph nodes) and in spinal cord of all L-BSE orally administered goats. The submandibular lymph nodes (LNs) demonstrated seeding reactions in four out of four L-BSE goats tested with an average lag phase of 36 h, whereas mesenteric, mediastinal and retropharyngeal lymph nodes showed seeding activity in 5/5, 3/4, 3/4, respectively, with longer lag phases (Fig. 2). Lower seeding activity was also detected in tonsils of all examined animals (5/5) (data not showed). RT-QuIC analysis of spinal cord also revealed PrP^sc^ deposition in the thoracic segment of 3/5 animals with stronger fluorescence increase for G2 goat. Goat G3 (see Table 1) produced a weakly positive signal in lumbar and sacral gray matter samples. No seeding activity was revealed in cervical gray matter. Further, CSF analyses by second generation of RT-QuIC, performed as previously described [5], did not detect PrP^sc^ in five out of five goats months after oral L-BSE administration. CSF from goat affected with L-BSE was used as positive control and showed strong fluorescence increase in the first hours of reaction.

## Discussion and conclusions

Data here presented indicate that caprine species are susceptible to L-BSE after oral administration and are able to produce very low levels of prions in both lymphatic and central nervous tissues as demonstrated by optimized, high-sensitive, RT-QuIC assay.

At variance with goats intracerebrally infected with L-BSE [4], in this study, no animal developed clinical signs of disease despite prolonged periods of observation, suggesting a comparatively low efficiency of the oral route versus the intracerebral one in L-BSE, a feature that further distinguish this strain from classical BSE [14, 15].

Interestingly, all goats tested negative by standard diagnostics for PrP^sc^ performed on brainstem. This finding, associated with the low amount of PrP^sc^ detected in different brain areas, suggests a partial strain-specific transmission barrier. Indeed, inoculation of a prion into a new host species can produce prolonged incubation periods and/or subclinical infection [16, 17]. In addition, the lack of clinical signs suggests that naturally L-BSE-infected goats may be asymptomatic similarly to what proposed by Okada et al. for oral L-BSE in cattle [17].

In line with previous results [18], RT-QuIC detected lower levels of prions than traditional diagnostic tools. Rapid and confirmatory tests failed to identify any PrP^sc^ in the subclinical animals, while RT-QuIC allowed us to detect misfolded prion protein in multiple brain regions, spinal cord and lymphoreticular system. Studies have established that the rate of fluorescence increase in RT-QuIC, while not measuring infectivity, is directly related to the concentration of prions in the sample seeding the reaction [19, 20]. Prolonged lag phases of RT-QuIC reactions indicate relatively low amounts of PrP^sc^ in the examined tissues and may reassure about the possibility of goat to play as silent L-BSE spreaders in natural conditions. However, we believe that prudence must be always adopted when dealing with the risk of prion spread in field conditions as also suggested by recent data by Denkers and colleagues, who showed that the oral route of infection for chronic wasting deisease in deer, may be much more efficient than previously thought [21]. Furthermore, although the mere presence of PrP^sc^ is not indicative of a possible infectivity of the tissue, the finding of positivity in the lymphoreticular tissue must alert to the potential distribution of PrP^sc^ in peripheral body regions which may increase the risks for humans. Bioassay of infectivity by inoculation of susceptible animals with brains of these goats may help to clarify this issue.

Based on the results achieved with this prion form and also other animal strains, it would be useful to consider the possibility to enlarge current diagnostic criteria to include, in defined conditions (e.g. very limited amounts of source tissue, or preclinical testing), the application of ultrasensitive diagnostic methods. This will not only improve the sensitivity of our surveillance systems but will also help to protect food chain from accidental spillovers of the agent of L-BSE.

## Limitations

The primary limitation of this work is that infectivity was not demonstrated by bioassay and the infectious titre was not determined. Therefore, we cannot comment the degree of risk for human.

Despite these limitations, this work specifically demonstrates prion-seeding activity in tissues of goats orally exposed to L-BSE and provide RT-QuIC as useful method to enhance surveillance of TSEs.

## Data Availability

The datasets used in the current study are available from the corresponding author by request.

## Abbreviations

BSE: Bovine Spongiform Encephalopathy
TSEs: Transmissible Spongiform Encephalopathies
L-BSE: L-type Bovine Spongiform Encephalopathy
PrPsc: Pathogenic Prion Protein
RT-QuIC: Real-Time Quaking Induced Conversion
CNS: Central Nervous System
LRS: Lymphoreticular System
PBS: Sterile Buffered Saline
CSF: Cerebrospinal Fluid
H&E: Haematoxylin and Eosin
ELISA: Enzyme-linked immunosorbent assay
IHC: Immunohistochemical
mAb: Monoclonal antibody
BHs: Brain Homogenates
THs: Tissues Homogenates
Ha-S: Hamster-Sheep
rPrPSen: Recombinant prion protein
LNs: Lymph Nodes
wt/vol: Weight/volume
SDS: Sodium Dodecyl Sulfate
ThT: Thioflavin T
EDTA: Ethylenediaminetetraacetic acid
CEA: National reference Center for Animal Encephalopathies
